# Hypohidrotic ectodermal dysplasia and Familial Mediterranean Fever in a child with recurrent episodes of hyperthermia

**DOI:** 10.1186/1546-0096-13-S1-P119

**Published:** 2015-09-28

**Authors:** L Guazzarotti, M Carrabba, S Beretta, M Zarantonello, I Sani, GV Zuccotti, G Fabio

**Affiliations:** 1 Ospedale Luigi Sacco & Università degli Studi di Milano, Pediatrics, Milano, Italy; 2 Fondazione IRCCS Ca’ Granda Ospedale Policlinico, Internal Medicine, Milano, Italy; 3 Università degli Studi di Milano, Clinical Sciences and Community Health, Milano, Italy; 4 Università degli Studi di Firenze, Scienze Biomediche, Sperimentali e Cliniche Mario Serio, Firenze, Italy

## Introduction

Ectodermal dysplasia (ED) is a clinically heterogeneous condition characterized by the abnormal development of two or more ectoderm-derived structures. Mutations in ED1 gene, (Xq12-13.1), are the most frequent cause. X-linked Hypohidrotic Ectodermal Dysplasia (XL-HED) is characterized by association of sparse hair, abnormal or missing teeth and variable inability to sweat that may cause recurrent episodes of hypertermia in the first years of age.

Familial Mediterranean Fever (FMF) is an autoinflammatory autosomal recessive disorder characterized by recurrent attacks of fever and serosal inflammation caused by mutations in the Mediterranean fever gene (MEFV), on chromosome 16p13.3.

An Italian child with clinical and molecular diagnosis of both conditions is described.

## Clinical report

The patient is the third child of unrelated parents. Dry skin with scaly lesions on trunk and limbs were noted at birth. At eight months of age they showed recurrent episodes of hyperthermia with normal inflammatory indices (ESR, CRP). At the age of 12 months, he showed the highly typical phenotype of HED and the molecular analysis of the ED1 gene was performed.

At the age of 32 months the child started presenting recurrent episodes of hyperthermia, colicky abdominal pain and abdominal rigidity at palpation with alterations of inflammatory indices and spontaneous resolution in 24 hours. Serum amyloid-A was: 808 mg/L (r.v.: < 6.40mg/L). Suspecting FMF, molecular analysis of the MEFV gene was performed.

## Methods and results

XL-HED was confirmed in the proband by direct sequencing of the entire coding region of the *EDA* gene and multiplex ligation-dependent probe amplification analysis to exclude genomic rearrangements, showing a missense mutation on exon 3 R156H (Arg156His), absent in the mother. FMF was confirmed in the proband by direct sequencing of the exons 2,5,10 showing the presence of three missense mutations: on exon 2 E148Q (p.Glu148Gln), on exon 10 A744S (p.Ala744Ser) and R761H (p.Arg761Cys). The proband inherited from his mother the mutations E148Q and R761H, and from his father the mutation A744S.

## Conclusions

ED are seldom associated to immunedysregulation. In our patient XL-HED and the disorder of the innate immune system co-exist for the association of two different gene mutations. The diagnosis of FMF is usually unproblematic in the presence of typical symptoms, however in our patient, the diagnosis was more challenging because of the overlapping symptoms of the two diseases. Molecular analysis played an essential role and allowed to start specific therapy with resolution of symptoms.

